# Supplementary figures and images for: Heterogeneous synaptic homeostasis: A novel mechanism boosting information propagation in the cortex
Source: PLoS Comput Biol. 2025 Aug 18;21(8):e1013398. doi: 10.1371/journal.pcbi.1013398 (PMC12385452; doi:10.1371/journal.pcbi.1013398)

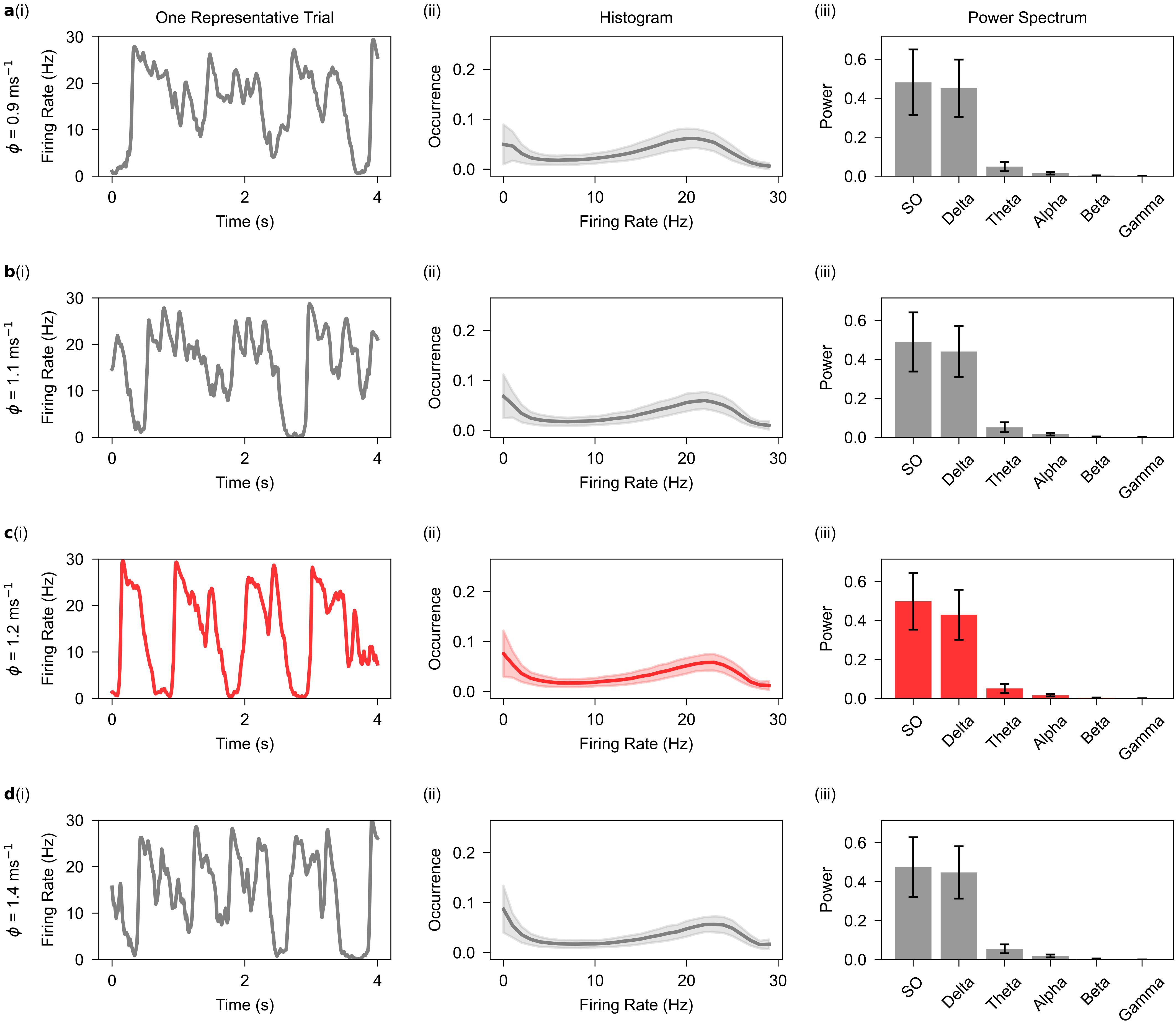

Supplement: S1 Fig — a, Spontaneous firing rate signal for a representative trial (i), the distribution of firing rate signals (ii), and the power spectrum of signals (iii) when ϕ=0.9 ms−1. b, c, and d, As in a, but for when ϕ increases. Panel c here is as Fig 1c. Note that ϕ=1.2 ms−1 is used as the value of the standard deviation of the noise in this computational study. Shaded area and Error bar correspond to standard deviation over 500 trials. (TIF) [file pcbi.1013398.s001.tif]

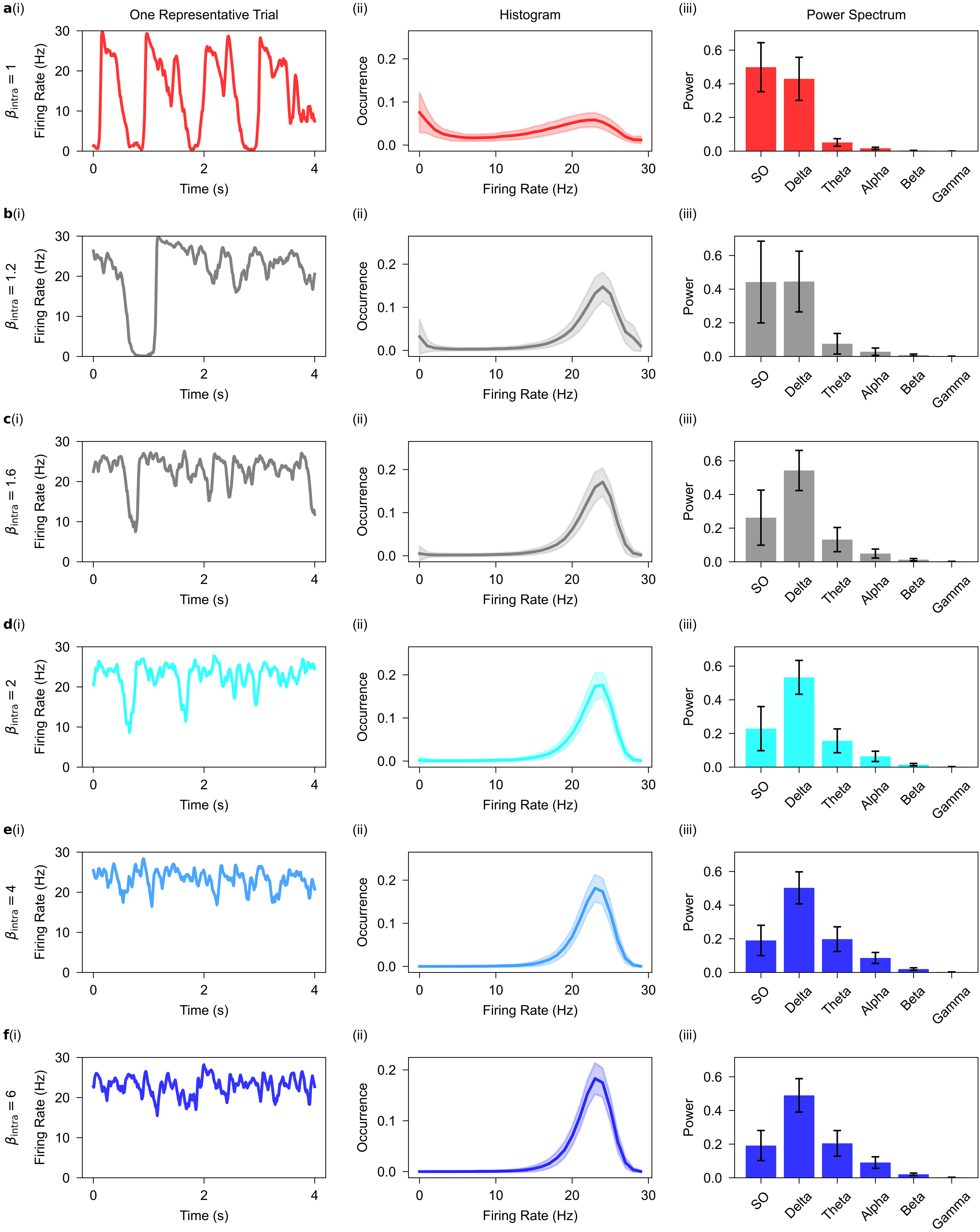

Supplement: S2 Fig — a, Spontaneous firing rate signal for a representative trial (i), the distribution of firing rate signals (ii), and the power spectrum of signals (iii) when there is no intra-synaptic upscaling (βintra=1). Panel a here is as Fig 1c. b, c, d, e, and f, As in a, but for when intra-synaptic upscaling (βintra) increases. Panel d here is as Fig 1d. Shaded area and Error bar correspond to standard deviation over 500 trials. (TIF) [file pcbi.1013398.s002.tif]

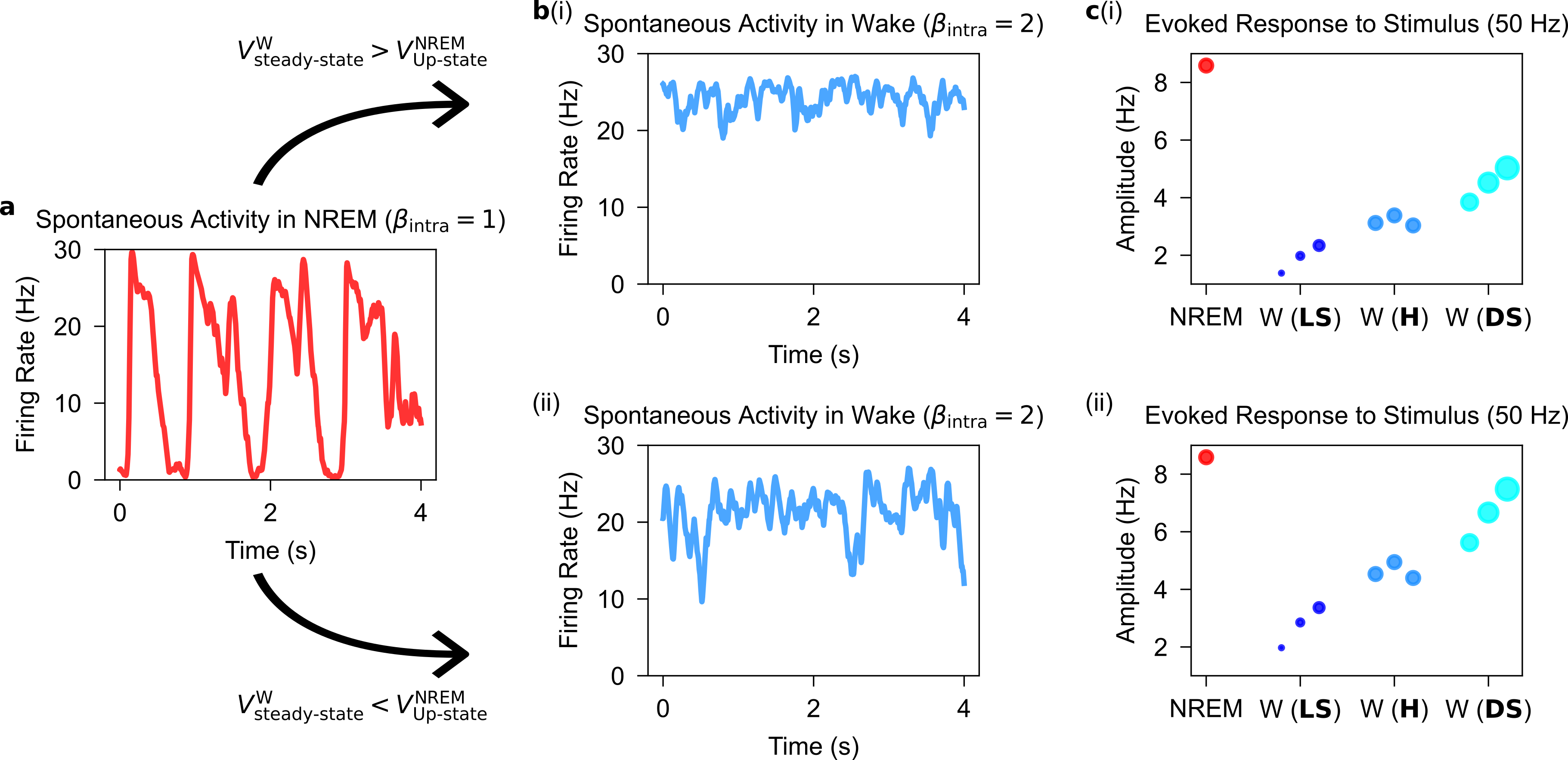

Supplement: S3 Fig — a, Spontaneous firing rate signal for the representative trial shown in Fig 1c(i). b, Same as in a, but with intra-excitatory connections upscaled (βintra=2). The steady-state membrane potential is set either higher (i) or lower (ii) than the Up state value during NREM sleep (red dashed horizontal line) by adjusting inhibitory synaptic strength βGABAk below and above the values shown in Table 5, respectively. c, Amplitude of evoked firing responses as a function of the synaptic upscaling ratio, βinter/βintra, during wakefulness. Note that regardless of whether the steady-state membrane potential is fixed at values either higher (i) or lower (ii) than the Up state during NREM sleep, we reproduce the behavior shown in Fig 2e. (TIF) [file pcbi.1013398.s003.tif]

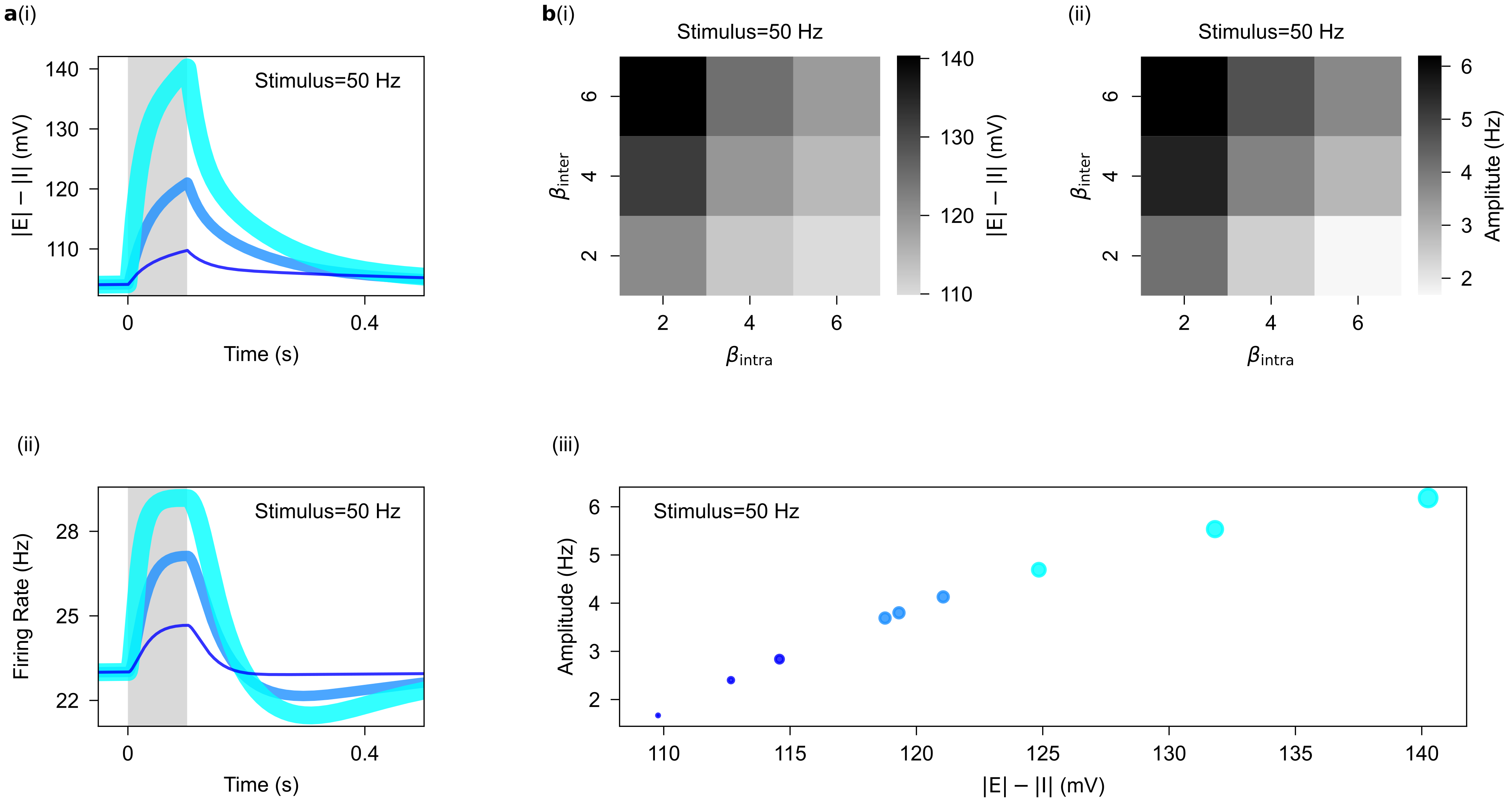

Supplement: S4 Fig — a, The net evoked synaptic current (i), quantified as |E|−|I|, decreases with increasing βintra as opposed to when βinter increases in wakefulness. The line width reflects the synaptic upscaling ratio, βinter/βintra. Changes in the time trace of net evoked synaptic currrent determine changes in the time trace of evoked firing responses (ii). Note that the net synaptic current remains constant before stimulus onset across various synaptic upscaling scenarios, illustrating that synaptic upscaling is implemented in a configuration without causing predominant excitation or inhibition. Shaded area corresponds to the stimulus duration. b, Effects of βintra and βinter on the net evoked synaptic currents explain the pulling and driving effects associated with the intra- and inter-synaptic upscalings in wakefulness. Intra-synaptic upscaling decreases the net evoked synaptic current (i) that results in the decreased evoked responses (ii). Conversely, inter-synaptic upscaling increases the net evoked synaptic current (i) that results in the increased evoked responses (ii). Changes in the net evoked synaptic currrent determine changes in the amplitude of evoked firing responses (iii). Note that analysis in b(i) are carried out on the data points at stimulus offset. (TIF) [file pcbi.1013398.s004.tif]

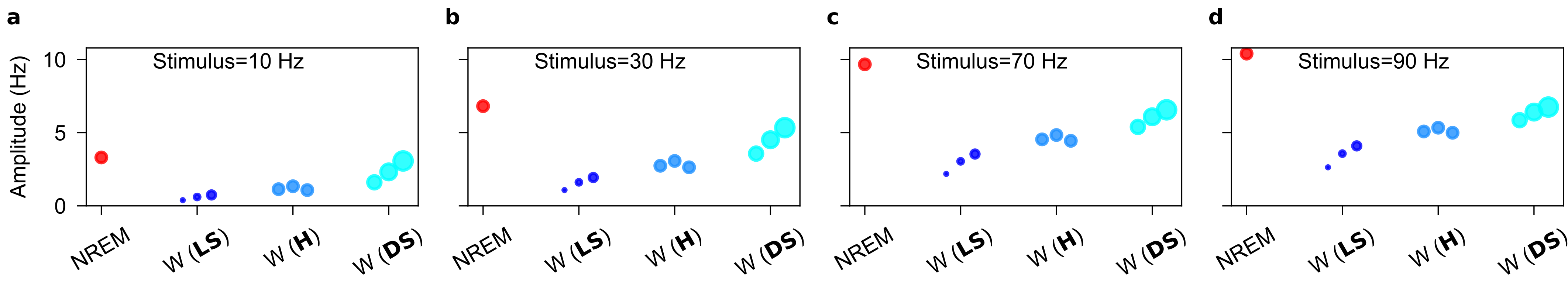

Supplement: S5 Fig — a, The amplitude of evoked firing responses increases with increasing values of synaptic upscaling ratio, βinter/βintra, during wakefulness when the stimulus intensity is 10 Hz (a), 30 Hz (b), 70 Hz (c) and 90 Hz (d). Note that the overall enhancement of the amplitude of evoked responses as the stimulus intensity increases from a to d. (TIF) [file pcbi.1013398.s005.tif]

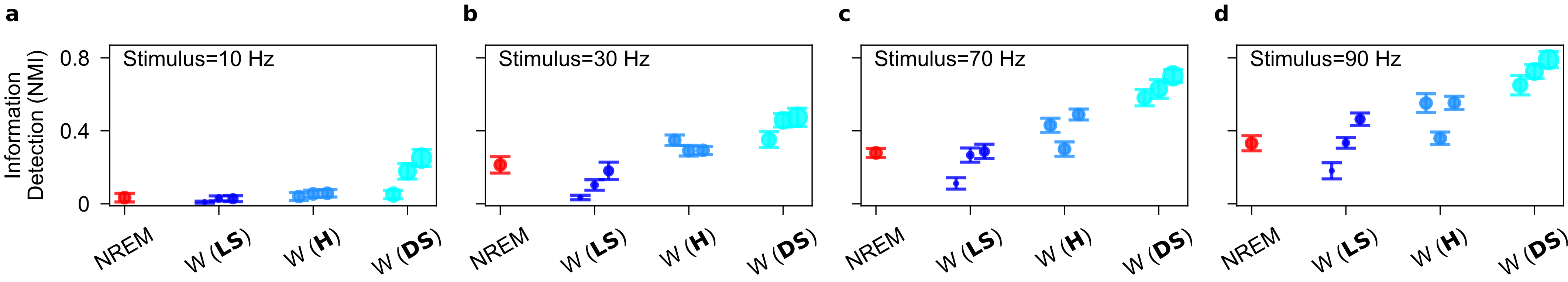

Supplement: S6 Fig — a, Information detection increases with increasing values of synaptic upscaling ratio, βinter/βintra, during wakefulness when the stimulus intesity is 10 Hz (a), 30 Hz (b), 70 Hz (c) and 90 Hz (d). Synaptic upscaling during wakefulness does not enhance information detection during wakefulness across stimuli compared to those in NREM sleep unless it occurs in DS upscaling. Note that the overall enhancement of information detection as the stimulus intesity increases from a to d. Error bar corresponds to 95% confidence interval over 10 performance estimate of the K-means clustering algorithms. (TIF) [file pcbi.1013398.s006.tif]

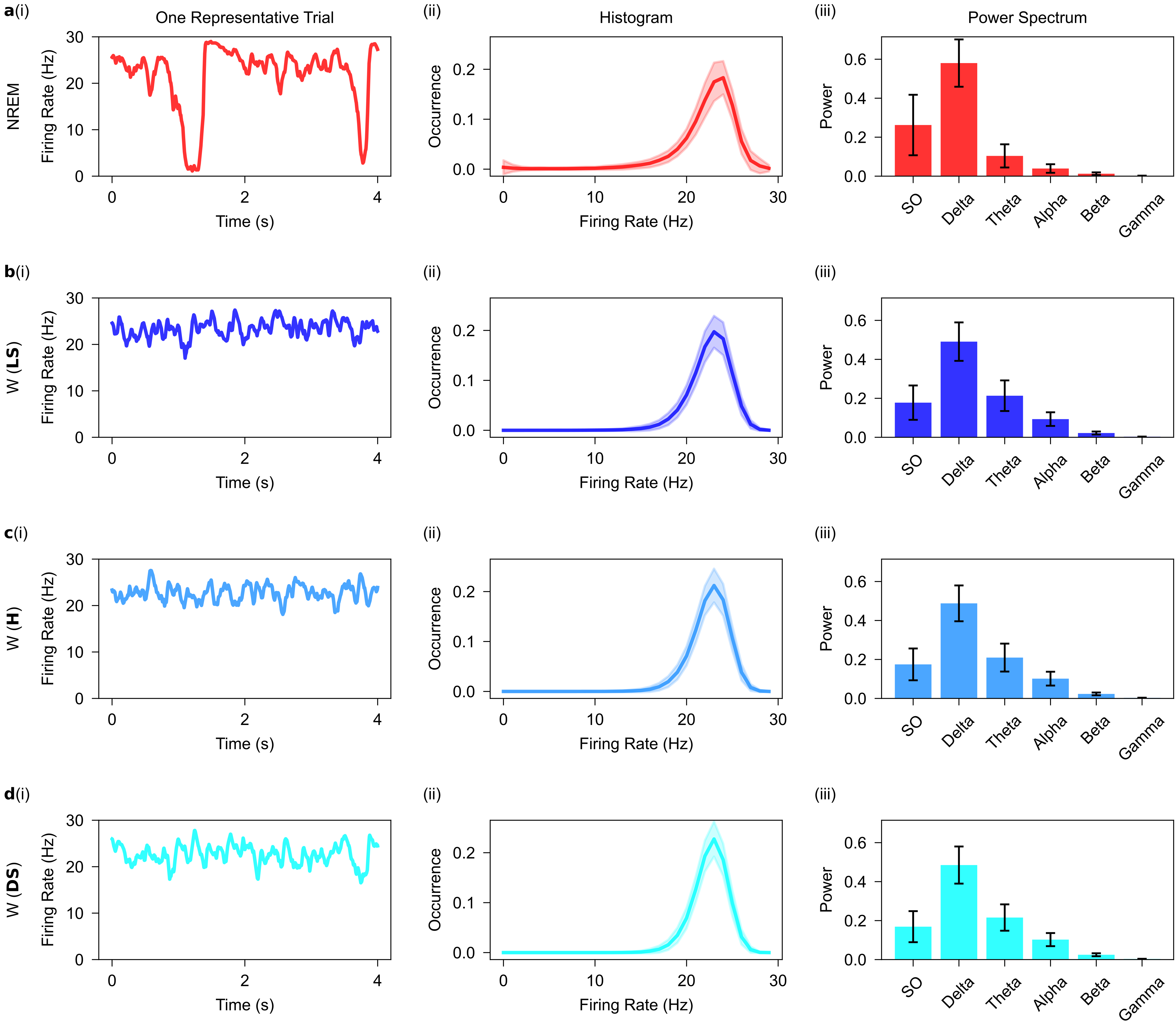

Supplement: S7 Fig — a, Spontaneous firing rate signal for a representative trial (i), the distribution of firing rate signals (ii), and the power spectrum of signals (iii) when there is no synaptic upscaling (βintra=1, βinter=1). b, c, and d, As in a, but for when synaptic upscaling is local-selective (LS: βintra=4, βinter=2), homogeneous (H: βintra=4, βinter=4), and distance-selective upscaling (DS: βintra=4, βinter=6), respectively. The dynamical features of spontaneous firing activity in the two-cortical-column model shift from NREM sleep to wakefulness for all synaptic upscaling combinations. Shaded area and Error bar correspond to standard deviation over 500 trials. (TIF) [file pcbi.1013398.s007.tif]

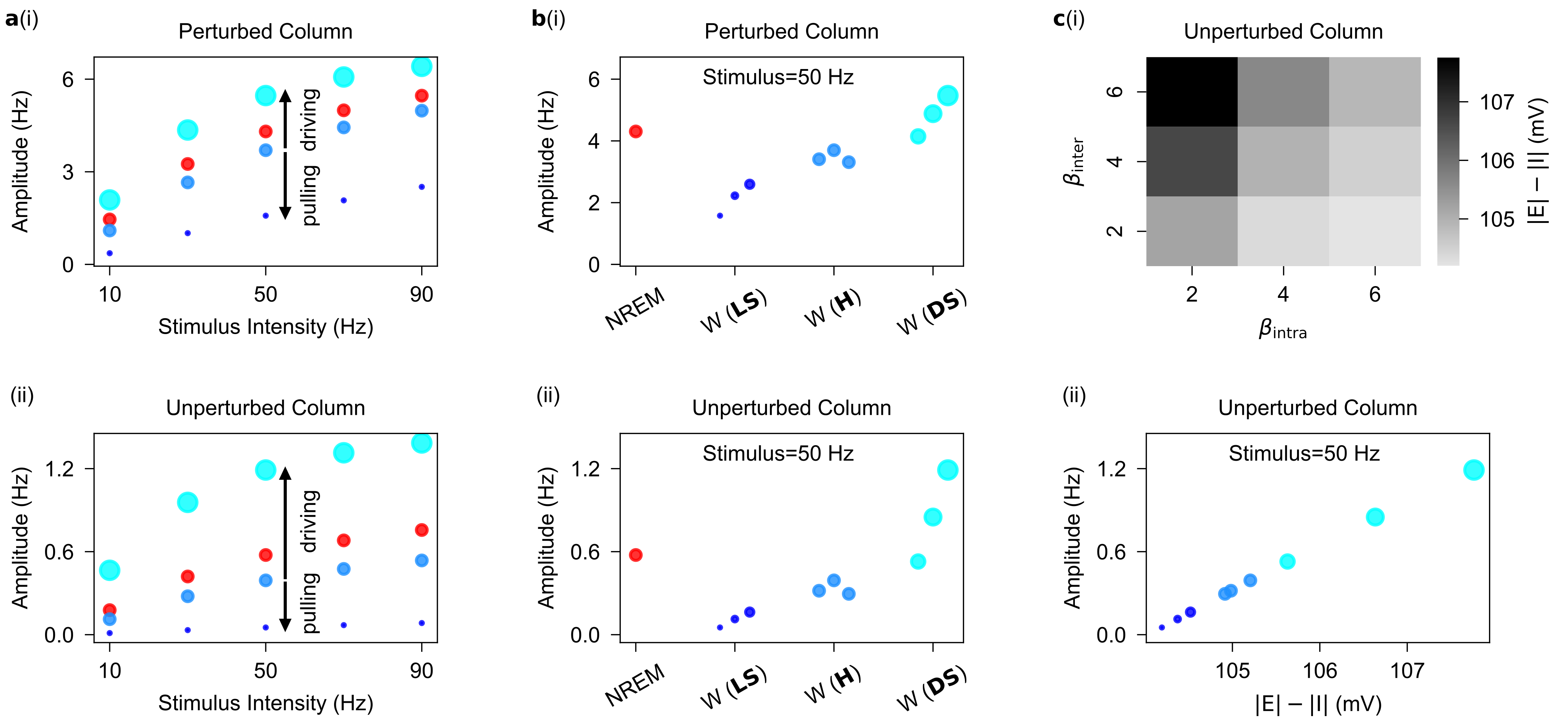

Supplement: S8 Fig — a, Increasing intra-synaptic upscaling while inter-synaptic upscaling is constant (from βintra=2,βinter=2 to βintra=6,βinter=2) during wakefulness produces a pulling effect on the amplitude of evoked firing responses in the perturbed (i) and unperturbed cortical column (ii). Conversely, increasing inter-synaptic upscaling while intra-synaptic upscaling is constant (from βintra=2,βinter=2 to βintra=2,βinter=6) during wakefulness produces a driving effect on the amplitude of evoked firing responses in the perturbed (i) and unperturbed cortical column (ii). b, The amplitude of evoked firing responses increases as the synaptic upscaling transitions from local-selective (LS) to distance-selective (DS) upscaling during wakefulness in the perturbed (i) and unperturbed cortical column (ii). Note that this holds true for other values of stimulus intensity. The amplitude of evoked responses to stimuli in the perturbed (i) and unperturbed cortical column (ii) during wakefulness enhances as synaptic upscaling transition from local-selective (LS) towards distance-selective (DS) upscaling. c, Inter-synaptic upscaling increases the net evoked synaptic current, as opposed to when intra-synaptic upscaling increases during wakefulness in the unperturbed cortical column (i). Changes in the net evoked synaptic currrent determine changes in the amplitude of evoked firing responses (ii). (TIF) [file pcbi.1013398.s008.tif]

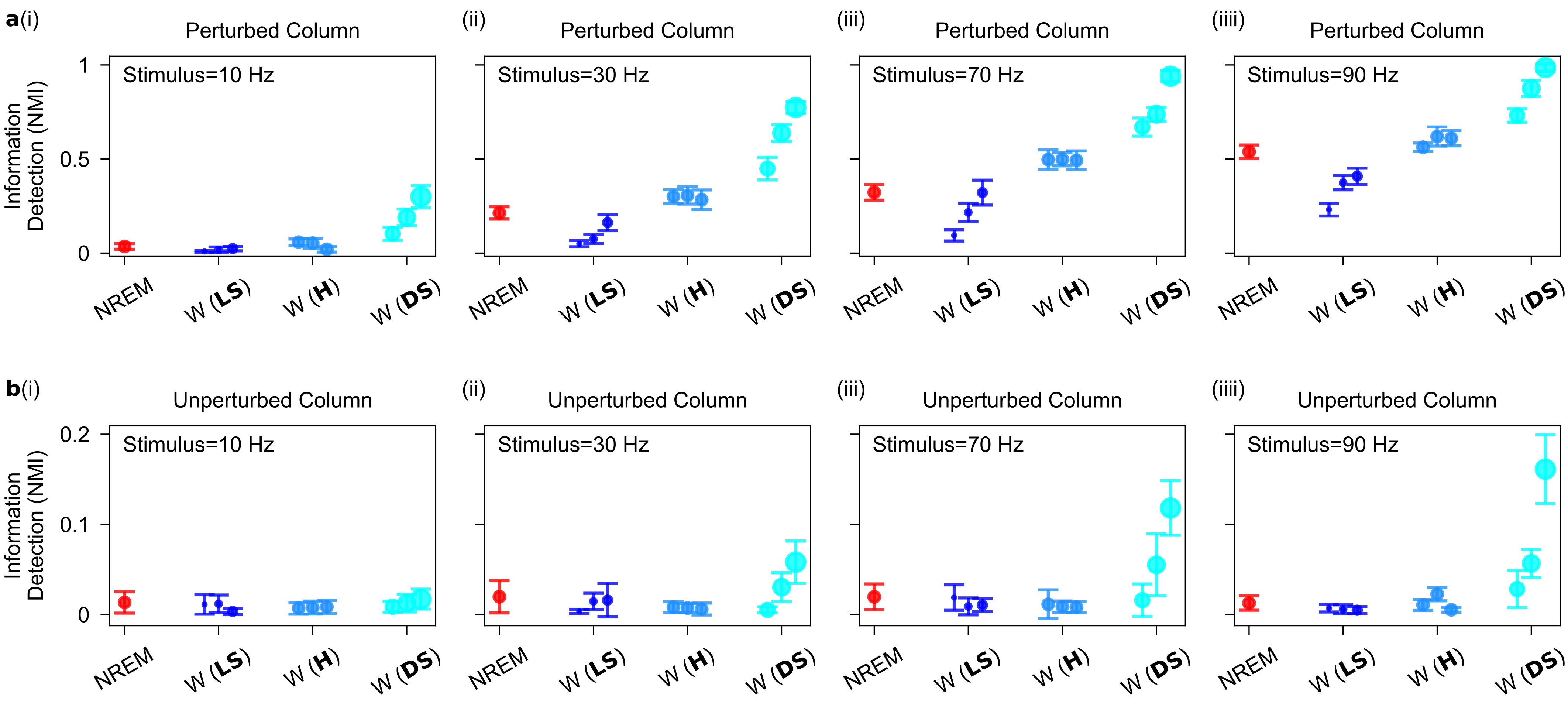

Supplement: S9 Fig — a, Information detection in the perturbed cortical column increases with increasing values of synaptic upscaling ratio, βinter/βintra, during wakefulness when the stimulus intesity is 10 Hz (i), 30 Hz (ii), 70 Hz (iii) and 90 Hz (iiii). b, As in a, but for the unperturbed cortical column. Error bar corresponds to 95% confidence interval over 10 performance estimate of the K-means clustering algorithms. (TIF) [file pcbi.1013398.s009.tif]

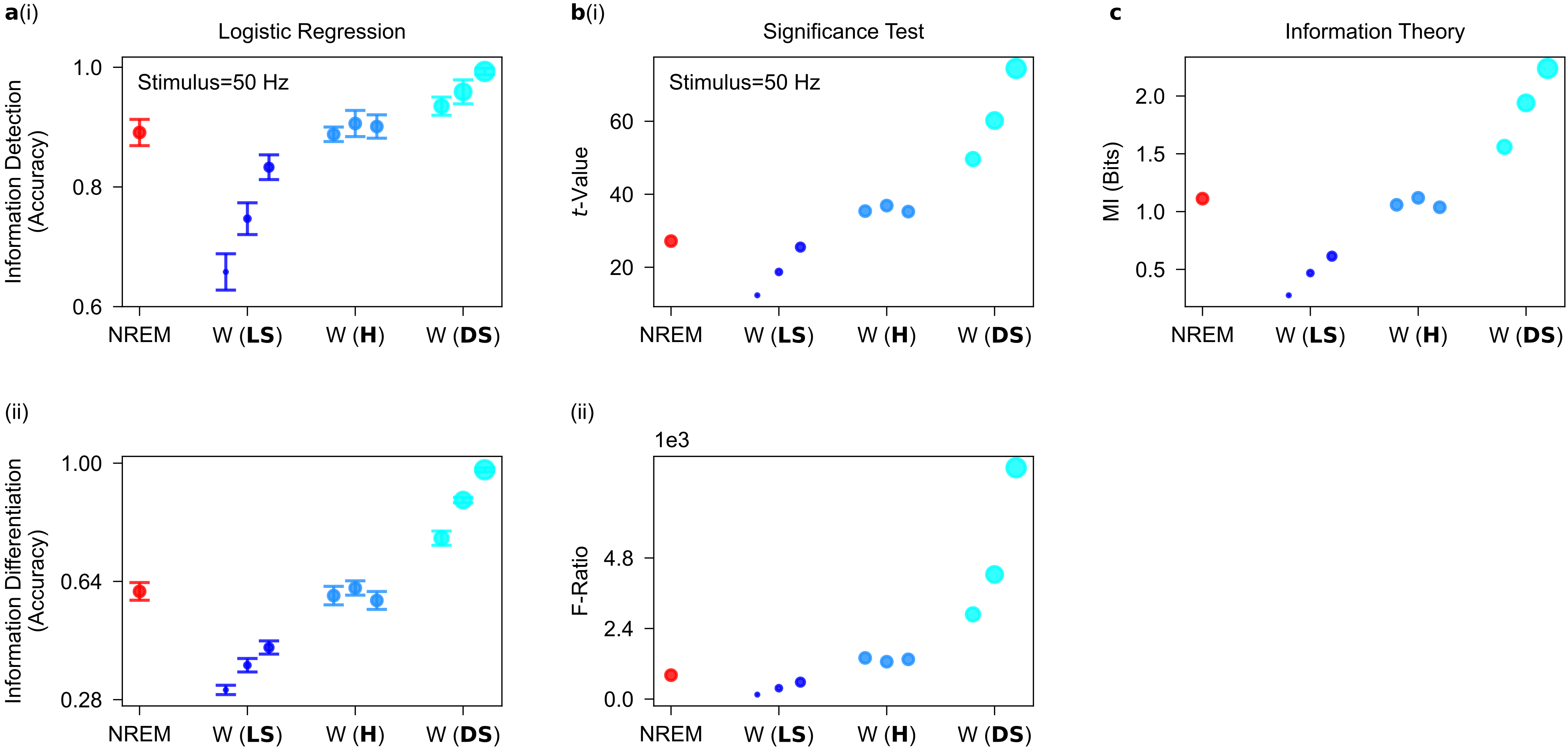

Supplement: S10 Fig — Figures pertain to analysis of evoked firing responses in the perturbed cortical column in the two-cortical-column model. a, Information detection for when stimulus intensity is 50Hz (i) and information differentiation (ii) when logistic classification algorithms (see S1 Appendix) are employed. Error bar corresponds to 95% confidence interval over 10 performance estimate of the logistic classification algorithms. Logistic classification algorithms qualitatively replicate the results obtained using K-means clustering algorithms in Fig 4 b. b, Implementing significance tests (see S1 Appendix) such as student t-test (i) and analysis of variance (ii) qualitatively replicate the results obtain by machine learning techniques pertaining to information detection and information differentiation. c, Implementing information theory (see S1 Appendix) manifests that the mutual information between the distribution of evoked responses at stimulus offset and the distribution of stimuli increases as synaptic upscaling transitions from local-selective (LS) to distance-selective (DS) upscaling during wakefulness. (TIF) [file pcbi.1013398.s010.tif]
