# Supplementary material for: Heterogeneous synaptic homeostasis: A novel mechanism boosting information propagation in the cortex
Source: PLoS Comput Biol. 2025 Aug 18;21(8):e1013398. doi: 10.1371/journal.pcbi.1013398 (PMC12385452; doi:10.1371/journal.pcbi.1013398)
Supplement: S1 Appendix — This conceptual framework was developed to evaluate stimulus-related information in neural signals using machine learning techniques, enabling the systematic extraction and quantification of information encoded in neural activity patterns. (PDF) [file pcbi.1013398.s011.pdf]

S1 Appendix: Information Quantification

Farhad Razi[1*], Belén Sancristóbal[2],

1 Donders Institute for Brain, Cognition and Behaviour, Radboud University, Nijmegen, The Netherlands
2 Computational Biology and Complex Systems Group, Department of Physics, Universitat Politècnica de Catalunya, Barcelona, Spain

* farhad.razi@donders.ru.nl

Information Encoding in Neural Signals

Neural activity can be spontaneous or stimulus-driven. In a simplified hierarchical processing model, neurons respond to external stimuli by generating firing patterns. These patterns then influence the activity of downstream neurons, creating a cascade of information flow [1, 2]. Postsynaptic neural spikes encode information about presynaptic firing, which in turn reflects the original external stimuli. We adopt the perspective that information, from a neuron's viewpoint, is *"a difference that makes a difference"* [3]. To quantify this information in neural responses, we introduce two novel measures: *information detection* and *information differentiation.*

Information detection

Information detection assesses whether stimulus-induced neural firing significantly differs from spontaneous activity. It determines if neural responses can be statistically linked to stimulus presentation. While crucial for perception, information detection alone doesn't guarantee rich stimulus encoding. For instance, if responses to two distinct stimuli both differ from spontaneous activity but not from each other, stimulus discrimination is limited. This scenario indicates attenuated, rather than absent, information content, as it still distinguishes stimulus-driven from spontaneous activity.

Information differentiation

Information differentiation evaluates whether neural responses to different stimuli are statistically distinguishable. It measures the ability to attribute neural firing to specific stimuli within a given set. The degree of differentiation may vary with the introduction of new stimuli. Together, information detection and differentiation determine the overall information content in neural responses.

High levels of both enable accurate stimulus feature decoding by an ideal observer with prior stimulus knowledge. This framework applies to both spike-based and rate-based theories, encompassing signal and noise correlation coding schemes.

Information propagation

Information flow between neural groups in a hierarchical processing chain can be assessed by measuring information content under different conditions. Consider a scenario where information content in a presynaptic group remains constant across two

brain states. If the postsynaptic group maintains information detection but loses differentiation in one state, it indicates attenuated information content and reduced information flow from presynaptic to postsynaptic neurons in that state. Both detection and differentiation can decrease (or increase) simultaneously during state transitions.

Information Quantification Metrics

To quantify information detection and differentiation, we employ machine learning algorithms. Information detection is measured by an algorithm's ability to distinguish stimulus-induced firing from spontaneous activity. Information differentiation is assessed by the algorithm's capacity to discriminate between responses to different stimuli. For researchers interested in replicating or extending our analysis, we have made our custom Python module for information quantification, *iQuanta*, publicly available on GitHub [4].

Unsupervised Machine Learning framework

Our unsupervised machine learning framework utilized the K-means clustering algorithm to quantify information in neural responses (see Materials and methods for details). For information detection, clustering was performed separately for each stimulus intensity and brain state to distinguish evoked from spontaneous activity at stimulus offset (see panel b in Fig 3). For information differentiation, K-means separated responses to different stimulus intensities within each brain state. Performance was measured using Normalized Mutual Information (NMI) [5] and 10-fold cross-validation [6], with results reported as average NMI and 95% confidence intervals (see panel c in Fig 3).

Supervised Machine Learning framework

In our supervised approach, we employed the Generalized Linear Model (GLM) to quantify information content in neural signals. This method involves three key steps: model selection, parameter estimation, and prediction [7]. The assumption of independent observations required by the GLM was satisfied through independent simulations across trials.

Information Detection: For information detection, we used a GLM with a Bernoulli distribution to model evoked firing responses at stimulus offset for a specific stimulus intensity, as well as spontaneous firing activities in each brain state. The GLM modeled the conditional distribution:

$$q(y \mid x, \theta)$$

where y is the response variable ($y = 0$ for absence of stimulus, $y = 1$ for presence of a given stimulus intensity), x is the firing rate of the pyramidal population, and θ are the model parameters. x consisted of 2×500 values (500 spontaneous firing rates and 500 evoked responses). We fitted a binomial logistic classification model to the training sets, optimizing parameters via the Newton-Raphson method to maximize the log likelihood [8, 9]:

$$\ell(\theta) = \sum_{i=1}^{n} [y_i \log p(x_i; \theta) + (1 - y_i) \log(1 - p(x_i; \theta))]$$

where $p(x_i; \theta)$ is the predicted probability for x_i. Model performance was assessed using stratified 10-fold cross-validation, with classification accuracy averaged across folds [6]. Higher accuracy values indicate greater information detection (see panel a(i) in S10 Fig).

Information Differentiation: To quantify information differentiation, we applied a GLM separately for each brain state, using a multinomial distribution to model evoked firing responses at stimulus offset across all stimulus intensities. Here, the response variable y took multiple values ($y \in \{0, 1, \ldots, N\}$ for N stimulus intensities), and x comprised $N \times 500$ firing rates (500 evoked responses per stimulus intensity). The multinomial log likelihood was maximized using the Newton-Raphson method:

$$\ell(\theta) = \sum_{i=1}^{n} \sum_{k=1}^{N} \mathbb{I}(y_i = k) \log p_k(x_i; \theta)$$

where $p_k(x_i; \theta)$ is the predicted probability for class k. $\mathbb{I}(y_i = k)$ is indicator function which equals 1 if the response variable y_i belongs to class k and 0 otherwise. Model performance was evaluated using stratified 10-fold cross-validation, with classification accuracy scores averaged across folds. Higher accuracy indicates greater information differentiation (see panel a(ii) in S10 Fig).

Significance Test

Moving beyond machine learning approaches, we performed statistical significance tests on the firing rate responses at stimulus offset to quantify changes in information content. To assess how significantly evoked responses at stimulus offset differ from spontaneous activities during prestimulus intervals, we employed Student's t-test separately for each stimulus intensity in both NREM sleep and all synaptic upscalings in wakefulness. Given the large number of trials ($n = 500$), satisfying the conditions of the Central Limit Theorem, we computed the independent t-test statistic (t-value) as follows:

$$t = \frac{\bar{x}_1 - \bar{x}_2}{s_p/\sqrt{n}}, \tag{1}$$

$$s_p^2 = \frac{(n-1)s_1^2 + (n-1)s_2^2}{2n-2} \tag{2}$$

Here, $\bar{x}_1$ and $\bar{x}_2$ represent the means across trials of evoked firing responses at stimulus offset to a given stimulus intensity and spontaneous firing activities at a random time point in prestimulus intervals, respectively. n is the number of trials, and s_1^2 and s_2^2 are the variances of x_1 and x_2 across trials. Statistical significance was set at $p < 0.05$. The t-value served as a measure of the distinction between evoked and spontaneous activities, with higher t-values indicating greater information detection (see panel b(i) in S10 Fig). To evaluate the distinctiveness of evoked responses across stimulus intensities, we conducted a one-way analysis of variance (ANOVA) separately for each brain state. The F-ratio was computed as:

$$F = \frac{\text{MSB}}{\text{MSW}}$$

where MSB and MSW are the between-group and within-group mean square values, respectively:

$$\text{MSB} = \frac{\sum_{i=1}^{N} n(\bar{x}_i - \bar{x})^2}{N-1}, \tag{3}$$

$$\text{MSW} = \frac{\sum_{i=1}^{N} \sum_{j=1}^{n} (x_{i,j} - \bar{x}_i)^2}{N(n-1)} \tag{4}$$

Here, N is the number of stimulus intensities, n is the number of trials, $x_{i,j}$ is the evoked firing response to stimulus intensity i in trial j, $\bar{x}_i$ is the mean response to

stimulus intensity i across trials, and $\bar{x}$ is the mean across all stimulus intensities. The F-ratio served as a measure of how distinct the evoked responses to different stimulus intensities were from each other, with higher F-ratios indicating greater information differentiation (see panel b(ii) in S10 Fig).

Information Theory

We also employed information theory to assess the informativeness of evoked firing responses at stimulus offset with respect to stimulus intensities. Mutual information quantifies the relationship between the distribution of firing rates and stimulus intensities [2]. Specifically, mutual information measures how much knowing the firing rate reduces uncertainty about a given stimulus, and is defined as:

$$I(X; s_j) = \sum_i p(x_i \mid s_j) \log_2 \frac{p(x_i \mid s_j)}{p(x_i)}$$

where $I(X; s_j)$ is the mutual information between the firing response distribution X and the given stimulus s_j. Here, $p(x_i)$ is the probability that the firing response takes the value x_i, and $p(x_i \mid s_j)$ is the conditional probability of observing x_i given stimulus s_j. Given that distribution of stimuli is uniform, summing the mutual information across all stimuli yields a scalar value, with greater separation among the distributions of evoked responses resulting in higher mutual information. Thus, higher mutual information values indicate greater information differentiation (see panel c in S10 Fig). For numerical computation, firing rates at stimulus offset were discretized into 0.1 Hz bins to estimate the required probabilities.

References

1. Gerstner W, Kreiter AK, Markram H, Herz AVM. Neural codes: Firing rates and beyond. Proceedings of the National Academy of Sciences. 1997;94:12740–12741. doi:10.1073/pnas.94.24.12740.

2. Borst A, Theunissen FE. Information theory and neural coding. Nature Neuroscience. 1999;2:947–957. doi:10.1038/14731.

3. Tononi G, Boly M, Massimini M, Koch C. Integrated information theory: from consciousness to its physical substrate. Nature Reviews Neuroscience. 2016;17:450–461. doi:10.1038/nrn.2016.44.

4. Razi F. Information Quantification; 2024.

5. Strehl A, Ghosh J. Cluster ensembles—a knowledge reuse framework for combining multiple partitions. Journal of machine learning research. 2002;3:583–617. doi:10.1162/153244303321897735.

6. Kuhn M, Johnson K. Applied predictive modeling. New York: Springer; 2013.

7. McCullagh P, Nelder JA. Generalized Linear Models. London: Routledge; 1983.

8. Bishop CM, Nasrabadi NM. Pattern recognition and machine learning. New York: Springer; 2006.

9. Boyd SP, Vandenberghe L. Convex optimization. Cambridge: Cambridge university press; 2004.
