# Supplementary material for: Heterogeneous synaptic homeostasis: A novel mechanism boosting information propagation in the cortex
Source: PLoS Comput Biol. 2025 Aug 18;21(8):e1013398. doi: 10.1371/journal.pcbi.1013398 (PMC12385452; doi:10.1371/journal.pcbi.1013398)
Supplement: Attachment — Submitted filename: FR_BS_PLOS_RebuttalLetter.pdf [file pcbi.1013398.s012.pdf]

Heterogeneous Synaptic Homeostasis: A Novel Mechanism Boosting Information Propagation in the Cortex
PCOMPBIOL-D-25-00090

We thank the reviewers for their insightful and constructive feedback.
We have revised the manuscript accordingly and we believe these changes have significantly strengthened our work. All modifications are highlighted in red in the revised manuscript.

Below, we address each comment in detail.

<u>Reviewer #1 Comments</u>

Comment 1: *Cell excitability can differ between wakefulness and the Up state during NREM sleep. The motivation behind calibrating the strength of inhibitory synapses during synaptic upscaling in wakefulness–so that the steady-state average membrane potentials of both pyramidal and inhibitory populations match their respective Up state values during NREM–is not entirely clear. Clarifying this point in the text would be helpful. Furthermore, including a supplementary figure demonstrating that the results are robust to variations in β_GABA would strengthen the analysis.*
Response 1: We appreciate this important point. Maintaining identical steady-state membrane potentials in both wakefulness and NREM Up states is motivated by experimental observations (Figure 9 in Steriade, M. et al. (2001); Figure 1A of in Destexhe, A et al. (2007); and Figure 2A in Constantinople, Ch. M. et al. (2011)). We have clarified this choice with the aforementioned references in the Materials and methods section (lines 129 – 137). To demonstrate robustness, we have included S3 Figure in the Supporting information, showing that evoked firing response patterns persist across a range of β_{GABA} that allow for different membrane potential values across the sleep-wake cycle (lines 135-137).

Comment 2: *Average firing rates during wakefulness are typically comparable to those observed during NREM sleep overall (see works from György Buzsáki), but are substantially lower than the firing rates during UP states within NREM. It would be interesting to know whether the conclusions of the present work would still hold if the model were adjusted to preserve this empirically observed relationship among firing rates across brain states.*
Response 2: We based our model on findings that overall firing rates in NREM sleep (including Down periods) are lower than during wakefulness (Vyazovskiy, V. V. et al., 2009). As noted by Ohyama, K et al., 2020 (see their Figure 1B), firing rates during Up states resemble those of wakefulness. We have clarified this in the revised text (lines 133-135). We also acknowledge György Buzsáki's findings on the decline in firing as Up states progress (Watson, B. O. et al. 2016) and have added a discussion of this dynamic in lines 138-142, although our study has not examined this feature of the dynamics. We did not find cortex-wide evidence of equal firing rates across brain states; we welcome specific references for this comment.

Comment 3: *Several figures are difficult to read due to missing legends and unclear parameter annotations. For example, in Figures 2d and 3a, it would be helpful to explicitly indicate the values of β_intra and β_inter within the figure panels. This suggestion extends to other relevant figures as well.*
Response 3: We agree with the reviewer and we have redesigned our figures for clarity. In Figure 2b, we summarize all parameters. The ratio of the synaptic-upscaling factor for inter-excitatory connections versus intra-excitatory connections, $\beta_{inter}/\beta_{intra}$, is now encoded by dot size. The upscaling policy is now coded by shades of blue: light blue corresponds to distance-selective (DS), blue to homogeneous-selective (HS) and dark blue to local-selective (LS). This has allowed to reduce the number of colors used and allows the reader to visually understand that the pulling effect correlates with a decrease in the size of the dots while the opposite occurs for the driving effect.

Comment 4: *To enhance self-containment and reproducibility, please consider including the mathematical definitions of the metrics used to quantify information detection and differentiation in the Methods section.*
Response 4: We agree that the Materials and methods section needs more details regarding such metrics and we have expanded the Unsupervised Machine Learning framework subsection, starting in line 242. Moreover, the code implementing this metrics is now fully available for the sake of transparency and reproducibility.

Reviewer #2 Comments

Comment 1: *Clarification of Statement on Stimulus-Evoked Propagation Patterns*
The authors state: "During wakefulness, neural responses to external stimuli exhibit a broader spatiotemporal propagation pattern compared to deep sleep." While this generalization is supported by several studies, it oversimplifies the complexity of stimulus-evoked dynamics across brain states. For example, evidence from [5] (2022, PNAS, https://doi.org/10.1073/pnas.2021252118) demonstrates that somatosensory stimulation during sleep can evoke bilateral activation in primary somatosensory cortices. The authors are encouraged to review and nuance this statement in the introduction to reflect a more accurate and comprehensive view of the literature.
Response 1: We thank the reviewer for this reference. We have nuanced this claim in the Abstract and Introduction (lines 5-9), citing Rosenthal et al. (2022) alongside Massimini, M. et al. (2007) to acknowledge wide cortical activation during anesthesia and sleep under certain conditions.

Comment 2: *Parameter Sensitivity and Generalizability of Results*
The model presented includes numerous parameters, and the outcomes may be highly sensitive to the specific values chosen. Although the authors base their parameter selection on previous literature (Tables 2–5), it remains unclear how robust the findings are to variations in these values. This does not undermine the validity of the proposed mechanism, but it does suggest that

different parameter configurations could produce varying outcomes—or that similar outcomes might arise from alternative configurations. Additionally, while the authors refer to the excitatory synapses as being in a "balanced" state, a clearer definition and explanation of how this balance is achieved would strengthen the study's clarity and reproducibility. The lack of direct experimental validation of the heterogeneous synaptic upscaling mechanism, while acknowledged, further emphasizes the need for caution in generalizing the findings.

Response 2: We agree with the reviewer that a common pitfall of these type of models if the choice of parameters and the robustness of the results to variations over other possible values. We now define "balance" explicitly as concurrent scaling of excitatory (β_{intra}) and inhibitory (β_{GABA}) intra-synaptic strengths to maintain a constant steady-state membrane potential (lines 71-75 and 129-135). A new supplementary S3 Figure shows evoked responses under over-excited (i) and over-inhibited (ii) wake-like states, confirming our main results hold across these conditions.

Comment 3: *Definition and Methodological Clarity for NMI*
Normalized Mutual Information (NMI) appears in the caption of Figure 3 before it is defined in the text. It is recommended that the term be introduced and explained prior to its use. While the authors commendably share the code for computing information detection and differentiation, these concepts should be thoroughly described in the methods section to ensure transparency and reproducibility.

Response 3: We thank the reviewer for having spotted this inconsistency and we have now introduced the NMI in lines 368-371, prior to its first mention in Figure 3's caption. We have also detailed its computation in the expanded Materials and methods (subsection Unsupervised Machine Learning framework, starting in line 242).

Comment 4: *Phase Diagram Enhancements and Reference Inclusion*
The phase diagram currently reports only the NMI values. It would be beneficial to also include information regarding the emergent dynamics of network activity in this space—specifically, the regions where slow oscillations occur. This would align the model more closely with empirical studies and facilitate interpretation. The authors are encouraged to cite and discuss related work, such as [1], which employs a similar analysis approach.

Response 4: We thank the reviewer for this suggestion and we have added a description of our stability analysis (lines 143-147) clarifying that both NREM-like and wake-like states are stable spirals, with wake-like dynamics having a dominant fast-decaying mode that suppresses oscillations.
We also discuss similarities with Tort-Colet, N, et al., 2021. Although in our case we have not modeled micro-arousal (MA) periods, but rather we have set our parameter values to simulate two distinct patterns of activities (NREM-like and wake-like activity) we can indeed smoothly increase the synaptic upscaling factor, i.e. β_{intra} and β_{GABA}, to sweep intermediate states where Down states gradually disappear (see S2 Figure) in close similarity with Figure 1B and 5D from Tort-Colet, N, et al., 2021. As a consequence, power within the slow band

decreases gradually from NREM-like to wake-like activity while delta power increases (in agreement with Figure 1D from Tort-Colet, N, et al., 2021). We have now emphasized these similarities between our work and the one suggested by the reviewer in lines 308-312.

Comment 5: *Literature Contextualization and Additional References*
The statement: "Most studies in sleep research have not explicitly evaluated information content and are primarily based on the amplitude of evoked neural signals," would benefit from appropriate citations to support this claim. The authors should distinguish between studies that focus on signal amplitude and those that address information content. To further strengthen the manuscript, the following references are recommended for inclusion due to their relevance to sleep-state dynamics and local/global network interactions:

[1] Tort-Colet, N., Capone, C., Sanchez-Vives, M. V., & Mattia, M. (2021). Attractor competition enriches cortical dynamics during awakening from anesthesia. Cell Reports, 35(12).
[2] Andrillon, T., & Oudiette, D. (2023). What is sleep exactly? Global and local modulations of sleep oscillations all around the clock. Neuroscience & Biobehavioral Reviews, 155, 105465.
[3] Miyazaki, T., Kanda, T., Tsujino, N., Ishii, R., Nakatsuka, D., Kizuka, M., ... & Yanagisawa, M. (2020). Dynamics of cortical local connectivity during sleep–wake states and the homeostatic process. Cerebral Cortex, 30(7), 3977-3990.
[4] Marsh, B., Navas-Zuloaga, M. G., Rosen, B. Q., Sokolov, Y., Delanois, J. E., González, O. C., ... & Bazhenov, M. (2024). Emergent effects of synaptic connectivity on the dynamics of global and local slow waves in a large-scale thalamocortical network model of the human brain. PLOS Computational Biology, 20(7), e1012245.
[5] Rosenthal, Z. P., Raut, R. V., Bowen, R. M., Snyder, A. Z., Culver, J. P., Raichle, M. E., & Lee, J. M. (2021). Peripheral sensory stimulation elicits global slow waves by recruiting somatosensory cortex bilaterally. Proceedings of the National Academy of Sciences, 118(8), e2021252118.
Response 5: We thank the reviewer for their thorough suggestion of related scientific literature. We have gone through it and corresponding citations have been made in the new version. In particular, reference [1] is cited in the context explained in Response 4 and in lines 147, 310 and 314. Reference [2] is cited in lines 314 and 522 in the context of local sleep. Reference [3] is now cited in line 531 to discuss evidence that sleep modulates local network connectivity, particularly during strong homeostatic regulation (as shown by their Figure 7). Reference [4] is now cited in line 76 in the context of sleep dynamics when connection strength is increased. Finally, reference [5] is cited in the context explained in Response 1 and in line 7.
Moreover, we have substantiated this claim: "Most studies in sleep research have not explicitly evaluated information content and are primarily based on the amplitude of evoked neural signals" by citing foundational event related potential studies in sleep research and highlight the novelty of using information-theoretic metrics (see lines 488-490).

Reviewer #3 Comments

Comment 1: *A more detailed dynamical systems analysis should be provided, at least for the one-column model. It appears that the awake-like activity is modeled as a stable node, while the NREM sleep-like activity is represented by a limit cycle driven by self-excitation and adaptation. This distinction should be clarified using standard linear stability analysis.*
Response 1: We agree with the reviewer that a detailed description of the stability analysis of our model is needed to prevent misunderstanding of the mathematical origin of the simulated dynamics. We clarify that both states, NREM-like and wake-like dynamics, correspond to stable spirals, not to a node or limit cycle, and that wake-like dynamics are distinguished by a strongly negative real eigenvalue leading to fast decay and suppressed oscillations. This clarification appears in lines 143-147.

Comment 2: *It is unclear whether the stimulation in the NREM sleep regime is applied during the upstate, the downstate, or if this distinction is not relevant. Clarification on this point would be helpful.*
Response 2: We agree with the reviewer that this has not been explicitly address in the manuscript. We now state explicitly that stimuli are applied at random times during NREM sleep, without conditioning on Up or Down states (lines 149-151).

Comment 3: *Could the authors comment on why they did not use Fisher information to quantify the discriminability between closely spaced stimuli? This seems like a natural choice for such an analysis.*
Response 3: Although it is true that Fisher Information (FI) can assess how well neural responses encode a stimulus variable, it is only valid when changes in a stimulus variable are small. Moreover, FI assesses how precisely we can estimate these small changes (stimulus intensity in our case) from a random variable (evoked firing rate responses in our case). In this study, we are using discrete values of stimulus intensity and from the set of simulated firing rates we are measuring how much information (in bits) they convey about the intensity. If we had modeled the intensity of the stimulus as a continuous variable around a particular value, we could have used FI to quantify precision of firing rates in encoding intensity.

Comment 4: *Finally, the authors should discuss their findings in relation to the work by Pachitariu et al. (2015, DOI: 10.1523/JNEUROSCI.3318-14.2015).*
Response 4: We agree with the reviewer that the suggested paper is very relevant to the topic of information encoding in the cortex across states. Although the authors use anesthesia as a proxy to study the slow oscillations of NREM sleep, it is true that our computational model cannot be ascribed to natural sleep. We now cite this paper (see lines 388-392) to emphasize the role of cortical dynamics in processing an external stimulus. In particular, and in agreement with our computational results, desynchronized activity (similar to our wakefulness state) is more responsive and selective to tone's frequency than the synchronized state (similar to our NREM-like dynamics).
